# Development and user experience of an innovative multi-mode stroke rehabilitation system for the arm and hand for patients with stroke

**DOI:** 10.1038/s41598-022-05314-8

**Published:** 2022-02-03

**Authors:** Yu-Wei Hsieh, Meng-Ta Lee, Chih-Chi Chen, Fu-Lin Hsu, Ching-Yi Wu

**Affiliations:** 1 Department of Occupational Therapy and Graduate Institute of Behavioral Sciences, College of Medicine, Chang Gung University, No. 259, Wenhua 1st Rd., Guishan Dist., Taoyuan City, 33302 Taiwan; 2 Department of Physical Medicine and Rehabilitation, Chang Gung Memorial Hospital, Linkou, 33305 Taiwan; 3 School of Medicine, College of Medicine, Chang Gung University, Taoyuan, 33302 Taiwan; 4 Healthy Aging Research Center, Chang Gung University, Taoyuan, 33302 Taiwan

**Keywords:** Health care, Neurology

## Abstract

Many individuals with stroke experience upper-limb motor deficits, and a recent trend is to develop novel devices for enhancing their motor function. This study aimed to develop a new upper-limb rehabilitation system with the integration of two rehabilitation therapies into one system, digital mirror therapy (MT) and action observation therapy (AOT), and to test the usability of this system. In the part I study, the new system was designed to operate in multiple training modes of digital MT (i.e., unilateral and bilateral modes) and AOT (i.e., pre-recorded and self-recorded videos) with self-developed software. In the part II study, 4 certified occupational therapists and 10 stroke patients were recruited for evaluating usability. The System Usability Scale (SUS) (maximum score = 100) and a self-designed questionnaire (maximum score = 50) were used. The mean scores of the SUS were 79.38 and 80.00, and those of the self-designed questionnaire were 41.00 and 42.80, respectively, for the therapists and patients after using this system, which indicated good usability and user experiences. This novel upper-limb rehabilitation system with good usability might be further used to increase the delivery of two emerging rehabilitation therapies, digital AOT and MT, to individuals with stroke.

## Introduction

Stroke is the leading cause of functional impairment and long-term disability among adults worldwide^1,2^. The incidence of stroke will increase to 35% in 2050 as the proportion of older adults rises^3^, and the cost and burden of post-stroke care remain substantial^2,4^. Four-fifths of stroke survivors experience arm and hand paresis with different degrees of severity^5^, and up to 66% of patients with affected upper limbs are still incapable of performing daily activities at 6 months post-stroke^6^. Thus, one of the top priorities in stroke rehabilitation is to develop and provide effective and specific interventions for improving upper-limb motor recovery and function in patients.

Over the last few years, mirror therapy (MT) and action observation therapy (AOT) have become promising approaches to enhance the efficacy of stroke motor rehabilitation^7^. In conventional MT, patients observe a reflection of the movements of the non-affected upper limb as if it were the affected one by using a plain mirror or mirror box^8,9^. Conventional MT is a simple, easy-to-use, and relatively low cost intervention^9^. During AOT, patients carefully observe motor acts in video clips and then physically practice the observed motor acts to the best of their ability^10^. Previous meta-analyses found that, compared with control interventions, AOT had significant small-to-moderate effects on arm and hand motor impairment and motor function^11,12^ and moderate-to-large effects on functional independence in basic activities of daily living^11^. Similarly, MT had significant medium effects on arm and hand motor impairment and motor function^9,13,14^ and small-to-moderate effects on functional independence in basic activities of daily living^9,14^, respectively.

Nevertheless, the use of a mirror box or a plain mirror in conventional MT may cause imbalances in trunk control and weight shifting, and a weak sense of body ownership, and the diversity of motor movements and tasks is limited^15,16^. Moreover, the pre-recorded video clips of AOT and the reflected visual illusions of MT in a mirror or mirror box may constrain the variety of therapeutic movements and functional tasks. In AOT, the pre-recorded video clips, especially those of functional tasks, may not be suitable for each individual patient’s needs; in MT, the limited space of the mirror box or the size of the mirror may restrict types of therapeutic tasks that the patients can practice.

Given the current advances in digital imaging technology, real-time video-captured images and computer-mediated visual feedback and stimuli have been developed and widely used in stroke rehabilitation^17–23^. Recently, some studies have recorded movements executed mainly by the non-affected hand of patients, immediately transformed the actions of the non-affected hand, and presented them on a screen or in goggles via cameras, webcams, or virtual reality technology^17,19,21,23–25^. This approach using real-time video-captured images broadens the diversity of movements and functional tasks and allows the users to record individual videos by themselves, which helps to overcome some limitations of conventional MT and AOT. These studies have also demonstrated the benefits of these devices or systems in improving upper-limb motor impairments in patients with stroke. Previous studies have found that computer-mediated visual illusions and real mirrored images produce similar degrees of neural activation^16,26^. Thus, computer-mediated visual feedback and stimuli (e.g., images or videos) may strengthen the clinical utility of MT and AOT.

Nowadays, for patients with stroke, computerized or digital MT devices focusing on upper-limb training have been widely developed with different technologies, such as digital imaging systems^25^, augmented reflection technology^19^, virtual-reality based equipment^23,24^, and camera-based mirror visual feedback^17^. These devices have the following individual advantages over conventional MT: (1) They minimize the tension of the cervical posture, asymmetry of the head and trunk, and weight shifting while the reflected images are viewed on a screen or in goggles^17,19,21,23,25^; (2) they increase the possibility of executing asymmetrical and reciprocal upper-limb movements^21,25^ or broadening the range of motion exercise and simulated real-life tasks^23,24^; and (3) they provide more vivid and convincing visual illusions when the reflected movements of the non-affected hand are directly superimposed on the affected hand on a computer screen^17,19^. In addition, some of them demonstrate clinical feasibility in stroke rehabilitation^17,19,21,23^. Although the space for performing all types of upper-limb movements or functional tasks^17^ may still be restricted or insufficient, computerized or digital MT devices show apparent promise.

With the evolution of digital imaging technology, it is now possible to extend and integrate the concepts of the two observational types of motor learning, conventional MT and AOT, to create a novel system/interface for wider clinical application. Integrating two effective and promising rehabilitation therapies, AOT and MT, into one digital system to achieve a more eclectic approach is needed for research and clinical use. This study aimed (1) to develop a new multi-mode stroke rehabilitation (MSR) system integrating digital AOT and MT and (2) to test the usability of this new system.

## Methods

### Part I: development of the MSR system

#### Hardware

**Figure 1:**
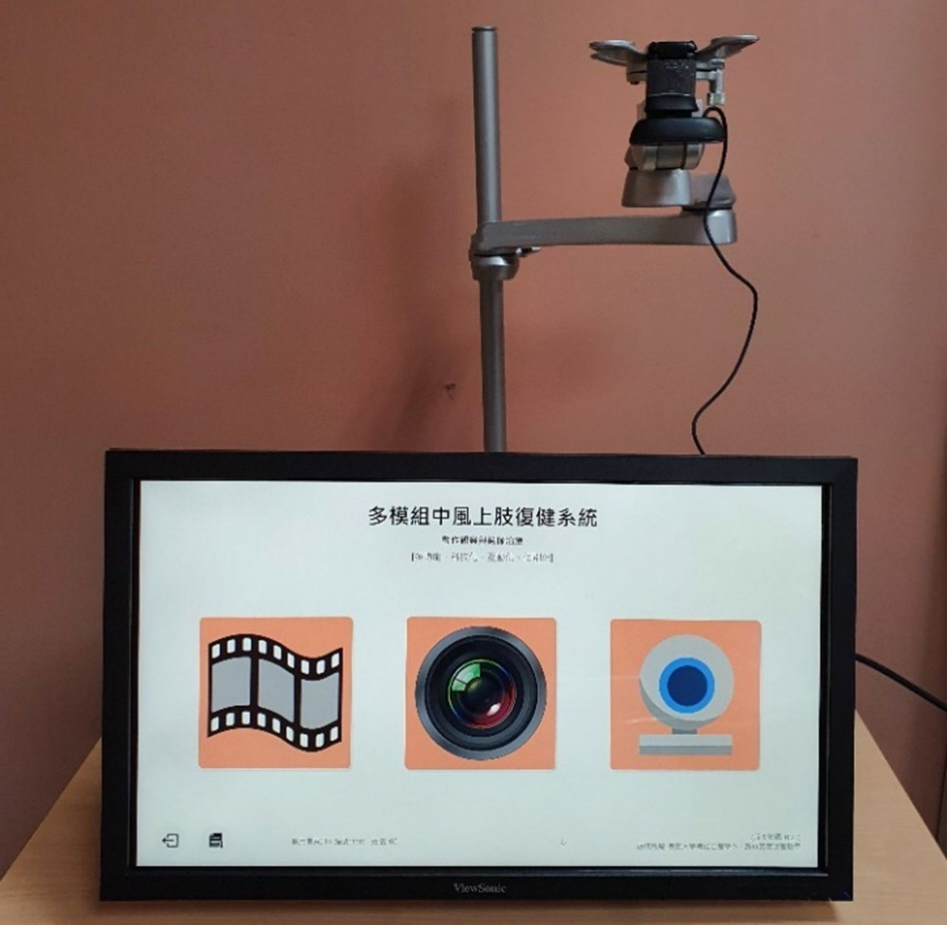
Interface of the multi-mode stroke rehabilitation system of arm and hand.

The hardware of the MSR system includes a laptop/personal computer, a computer screen, an adjustable monitor stand with two arms, and a webcam (Fig. 1). A 24-inch computer screen (1920 × 1080) is mounted in front of the user, attached to one arm of the adjustable stand, which allows the user to move the screen for conducting either digital MT or digital AOT. In addition, an HD webcam (resolution 1280 × 720, 60° field of view) is mounted to the other arm of the adjustable stand to capture the movements. The webcam is connected to the computer via a Universal Serial Bus (USB) port. The operating system can run on any low-end laptop meeting the minimum RAM requirement of 2 GB, a computer stick (i.e., a mini personal computer), and even a Chromebook that can execute a modern web browser (i.e., Chrome, Firefox or Safari).

During digital MT, the webcam is used to capture the movements of the patient’s non-affected hand, and then the video stream of the movements of the non-affected hand is processed to produce either a mirror image of the patient’s affected hand (i.e., unilateral mode) or that of both hands (i.e., bilateral mode) on the screen. The bilateral mode is a particular type of mirror visual feedback which differs from the type provided by conventional MT. Additionally, for digital AOT, the webcam is also used to record the actions and movements of the users to produce the video clips.

#### Software

The self-developed software is written in modern web programming languages, including HTML5, CSS, and JavaScript, so that the program can be robustly deployed into several clinical applications with convenient and flexible features. To efficiently extract the webcam stream to the browser, the RecordRTC library (v5.5.3) is applied to present a preview of the webcam and to record the webcam stream from the USB port of the computer. There is a very small and not easily noticeable/visible delay between the actual movements and the display of the transformed movements. An HTML5 element, canvas, is applied to administer multiple previews and video displays on a single webpage. The CSS transform property with the rotate method is applied to efficiently flip the preview/video horizontally. All the user interfaces and interactions were fabricated with HTML5/CSS/JavaScript art. The users’ data are stored in the “LocalStorage” of the browser and can be exported in csv and/or text formats for further research or clinical use. The data of the user information, the date and place of intervention, and the list of AOT videos watched can be exported. The exported data can be used as a brief intervention note. The software is designed for implementation as a localhost application via browser plugin (i.e., 200OK) to run a local server without connecting to the internet, which prevents personal data leakage and thereby improves data safety. Thus, users can implement and apply this system at home.

#### Training modes

**Figure 2:**
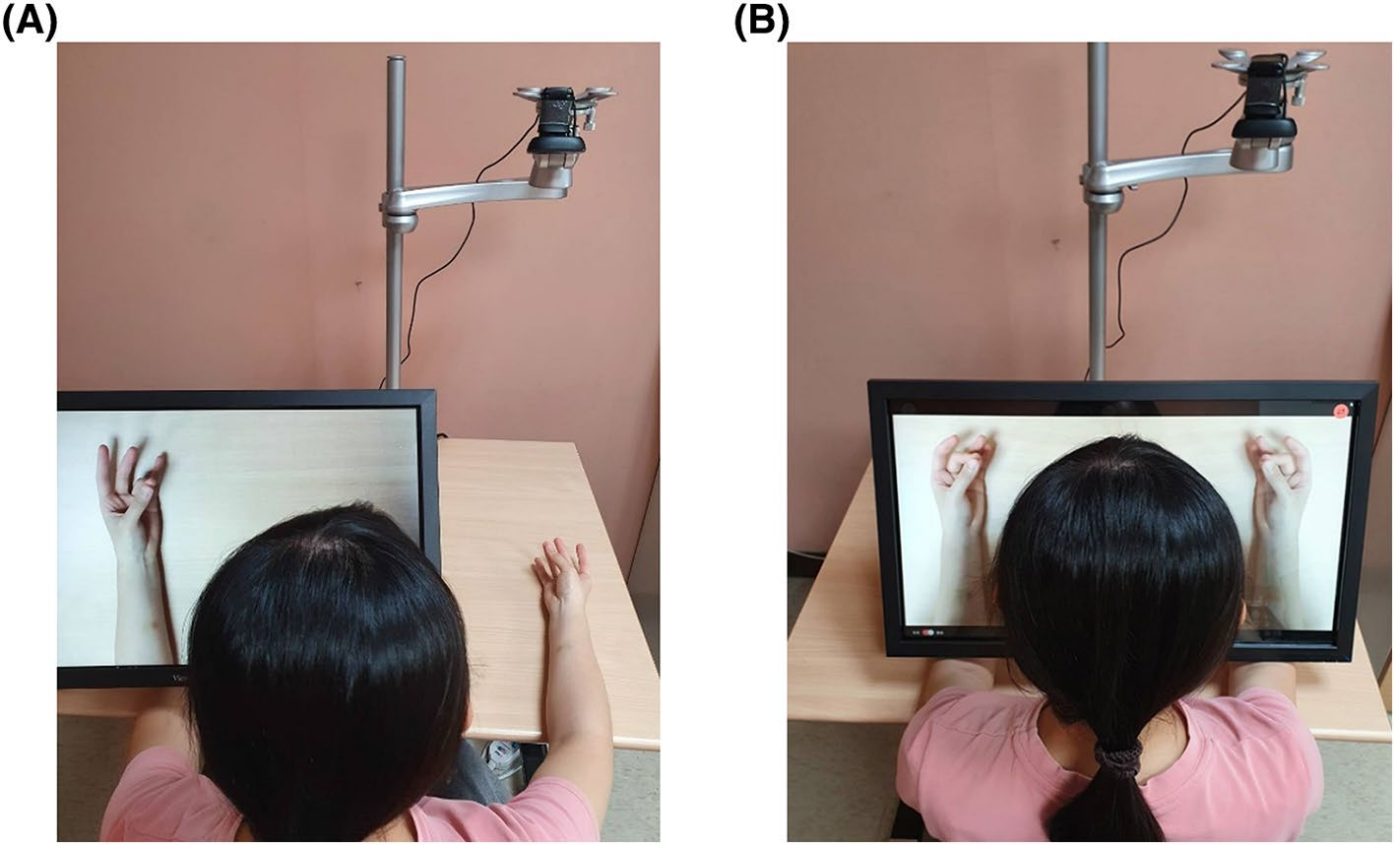
Demonstrations of digital mirror therapy: (**A**) unilateral mode, and (**B**) bilateral mode. (**A**) The movements of the user’s non-affected hand (e.g., right hand) can be recorded by the webcam, instantly transformed into mirror images of the affected hand’s movements (e.g., left hand), and presented on the screen by the self-developed software. (**B**) The movements of the patient’s non-affected hand can also be recorded, instantly transformed into mirror images of movements of both hands, and simultaneously presented on the screen.

The MSR system was designed to operate in multiple training modes of digital MT (i.e., unilateral and bilateral modes) and AOT (i.e., pre-recorded and self-recorded videos). For the 2 training modes of digital MT, the movements of the patient’s non-affected hand (e.g., right hand) can be recorded by the webcam, instantly transformed into mirror images of the movements of the affected hand (e.g., left hand), and presented on the screen by the self-developed software in the unilateral mode (Fig. 2A). The affected hand of the patient is placed under the screen and cannot be seen. In addition, the movements of the patient’s non-affected hand can also be recorded, instantly transformed into mirror images of both hands’ movements, and simultaneously presented on the screen in the bilateral mode (Fig. 2B). The patient is asked to watch the images of the movements presented on the screen and imagine that they are the movements of his/her affected hand or both hands. The attending therapist selects the most suitable mode or combines the use of different modes of mirror visual feedback to tailor interventions to meet the patient’s individual needs and therapy goals.

For the 2 training modes of digital AOT, the users can either select pre-recorded videos in the system or record the actions or movements by themselves (i.e., self-recorded videos) for action observation. The movements in the self-recorded videos can be performed either by the non-affected arm and hand of patient or by their therapist’s or caregiver’s upper limb. The pre-recorded videos contain active range of motion, object manipulation, reaching movements, grasping/releasing tasks and function-oriented tasks for providing different categories of movements or actions. Two phases, the action observation phase and the action execution (i.e., physical practice) phase, can be implemented during digital AOT.

### Part II: usability assessment of the MSR system

#### Participants

The participants, who were certified occupational therapists and patients with stroke, were recruited to participate in the usability assessment of this new system between August 2020 and March 2021. The inclusion criteria of the patients with stroke were (1) diagnosis of unilateral hemispheric stroke; (2) age of 20 to 80 years; (3) baseline score on the Fugl-Meyer Assessment of the Upper Extremity (FMA-UE) of 20–60^27,28^; and (4) ability to follow the instructions and provide user feedback verbally. Eligible patients with stroke were excluded if they had (1) global or receptive aphasia, evaluated by medical record, (2) severe neglect, assessed by the line bisection subtest in the Behavioral Inattention Test (mean deviations ≥ 1.5 inches on the 8-inch horizontal line), or (3) major medical problems or comorbidities that could influence upper-limb usage or cause severe pain.

#### Ethics declarations

This study was approved by the Institutional Review Board of the Chang Gung Memorial Hospital (201901885A3) and the Taipei Hospital, Ministry of Health and Welfare (TH-IRB-0020–0004). Written informed consent was obtained from the certified occupational therapists and patients with stroke before they participated in the study. All methods were performed in accordance with Declaration of Helsinki.

#### Study design and procedures

In this cross-sectional study, the demographic data of occupational therapists and patients with stroke were collected. The clinical data of the patients, such as stroke type, hemispheric side of lesion, and time since stroke onset, were also gathered. The main reason for including the therapists was that we also wanted to understand the perspectives of the therapists on using and operating this new system for their clients, as well as their views of its usability and suitability for clinical use. A well-trained researcher administered the FMA-UE to the patients.

During the testing, each participant used each training mode of digital AOT (i.e., pre-recorded and self-recorded videos) and MT (i.e., unilateral and bilateral MT) with the assistance of a researcher who was also a certified occupational therapist. After completion of the testing, the participants were asked to complete the System Usability Scale (SUS) and a self-designed questionnaire to assess the user experiences, their perspectives on this new MSR system, and their view of its suitability for stroke rehabilitation.

### Measurements

#### System Usability Scale (SUS)

The SUS, a 10-item questionnaire with a 5-point Likert-type scale ranging from 1 (strongly disagree) to 5 (strongly agree)^29^, is a reliable and validated measure for evaluating the subjective experience of using a technological system^30^. In this study, the SUS was used to assess the user’s experience and satisfaction with the MSR system. The 10 items of the SUS are as follows: (1) I think that I would like to use this system frequently, (2) I found the system unnecessarily complex, (3) I thought the system was easy to use, (4) I think that I would need the support of a technical person to be able to use this system, (5) I found the various functions in this system were well integrated, (6) I thought there was too much inconsistency in this system, (7) I would imagine that most people would learn to use this system very quickly, (8) I found the system very cumbersome to use, (9) I felt very confident using the system, and (10) I needed to learn a lot of things before I could get going with this system.

The odd-numbered items (# 1, 3, 5, 7, and 9) are positive items, whereas the even-numbered items (# 2, 4, 6, 8, and 10) are negative items. For odd-numbered items, the score is the given scale position minus 1; for even-numbered items, the score is 5 minus the scale point given by the user. Thus, for the odd-numbered items, a greater score indicates higher agreement with the statement of the item. However, for the even-numbered items, a lower score indicates higher agreement with the statement of the item. In addition, the total score of the SUS is transformed into 0 to 100 by summing the scores of the items and then multiplying the sum by 2.5. Higher scores indicate greater usability. SUS scores above 70 and 85 indicate good and excellent usability, respectively^30^.

#### Self-designed questionnaire

The self-designed questionnaire was a 10-item questionnaire with a 5-point Likert-type scale ranging from 1 to 5. It was used to assess the user experiences with the specific hardware components and arrangements of this MSR system, including (1) the concentration level of the participant while using this MSR system, (2) the placement of the screen, (3) the placement of the camera, (4) the height of the seat, (5) the design of the working platform, (6) the resolution of the videos and images, (7) the ease of operating the two adjustable arms, (8) the ease of use of the computer interface, (9) the ease of use of the user manual, and (10) willingness to use this MSR system in the future. The total score of the self-designed questionnaire ranged from 10 to 50. Higher scores indicated better user experiences.

### Statistical analysis

Descriptive statistics, such as mean ± standard deviation (SD), range, and frequency, were used to describe the demographic and clinical characteristics of the participants and to demonstrate the assessment of the SUS and self-designed questionnaire. Additionally, the percentages of the SUS rating scores above 70 and 85 respectively in occupational therapists and patients with stroke were also calculated.

## Results

### Usability assessment of the MSR system

#### Demographic and clinical characteristics of the participants

Four certified occupational therapists and 10 patients with stroke were recruited in this study. The occupational therapists had 4.17 to 19.83 years of clinical experience (mean ± SD: 12.06 ± 7.99), and 3 of them were female (Table 1). Two therapists had experience in using conventional mirror therapy (e.g., a mirror box) in their clinical practice.

**Table 1:** The demographic characteristics of the certified occupational therapists.

Subject	Clinical experience (years)	Sex
OT01	18.00	F
OT02	4.17	M
OT03	19.83	F
OT04	6.25	F
	Mean: 12.06 (SD: 7.99)	

Abbreviations: F, female; M, male; SD, standard deviation.

In addition, all the patients were right-hand dominant, and most of the patients were male (*n* = 8). The patients ranged in age from 30.17 to 71.83 years (mean ± SD: 52.41 ± 12.94), and the time since stroke onset ranged from 8 to 34 months. Six and 4 patients were respectively diagnosed as ischemic and hemorrhagic stroke, and there were 4 right and 6 left hemisphere lesions. The total FMA-UE scores of the patients ranged from 22 to 57 (mean ± SD: 38.20 ± 12.02), indicating moderate to mild upper-limb motor deficits (Table 2).

**Table 2:** The demographic and clinical characteristics of the patients with stroke.

Subject	Age (years)	Sex	Hand dominance	Brain lesion side	Stroke type	Time since stroke onset (months)	FMA-UE score (0–66)
PT01	33.25	M	R	R	I	31	34
PT02	61.75	F	R	L	I	32	33
PT03	48.92	M	R	L	I	18	32
PT04	71.83	M	R	L	H	16	57
PT05	53.50	M	R	L	H	34	33
PT06	58.25	M	R	L	I	15	55
PT07	60.92	M	R	L	I	25	26
PT08	30.17	F	R	R	I	17	50
PT09	58.25	M	R	R	H	8	22
PT10	47.25	M	R	R	H	23	40
	Mean: 52.41 (SD: 12.94)					Mean: 21.90 (SD: 8.54)	Mean: 38.20 (SD: 12.02)

Abbreviations: F, female; FMA-UE, Fugl-Meyer Assessment of the Upper Extremity; H, hemorrhagic; I, ischemic; L, left; M, male; R, right; SD, standard deviation.

Note: Higher FMA-UE scores are indicative of less upper-extremity motor impairment.

#### Usability of the MSR system

In general, the participants spent about 1 to 1.5 h using the MSR system in one day. During this time, they learned the functions of the system, learned how to operate the software and hardware of the system, and tested each training mode of digital AOT and MT. The participants spent about 40 to 50 min testing and executing the digital AOT and MT training modes and tasks. After the testing, they completed the SUS and self-designed questionnaire.

#### Therapist experiences and feedback

For the occupational therapists, the mean score of the SUS was 79.38 (Table 3). Three therapists and 1 therapist gave SUS scores above 70 and 85, respectively, which indicated good and excellent usability. Furthermore, for the therapists, the item of the SUS with the highest average score was “I felt very confident using the system”, and the item of the SUS with the lowest average score was “I think that I would need the support of a technical person to be able to use this system” (Table 4).

**Table 3:** Usability of the multi-mode stroke rehabilitation system in patients with stroke and occupational therapists.

	Occupational therapists (*n* = 4)	Patients with stroke (*n* = 10)
SUS score (0–100)	79.38 ± 12.81 (Range: 62.5–92.5)	80.00 ± 11.61 (Range: 60–100)
> 70	3 (75%)	8 (80%)
> 85	1 (25%)	2 (20%)
Self-designed questionnaire score (10–50)	41.00 ± 2.45 (Range: 38–44)	42.80 ± 5.85 (Range: 32–50)

The listed statistics are presented as mean ± standard deviation (SD) or frequency (percentage) as appropriate for the data type.

Abbreviations: SUS, System Usability Scale.

Note: Higher SUS scores are indicative of greater usability. Higher scores of the self-designed questionnaire are indicative of better user experiences.

**Table 4:** Mean ratings on each item of the System Usability Scale in occupational therapists and patients with stroke.

	Occupational therapists (*n* = 4)	Patients with stroke (*n* = 10)
1. I think that I would like to use this system frequently	3.25 ± 1.50 (Range: 1–4)	3.70 ± 0.48 (Range: 3–4)
2. I found the system unnecessarily complex	3.00 ± 0.82 (Range: 2–4)	3.30 ± 0.95 (Range: 1–4)
3. I thought the system was easy to use	3.50 ± 0.58 (Range: 3–4)	3.70 ± 0.67 (Range: 2–4)
4. I think that I would need the support of a technical person to be able to use this system	2.50 ± 0.58 (Range: 2–3)	2.00 ± 1.25 (Range: 1–4)
5. I found the various functions in this system were well integrated	3.25 ± 0.50 (Range: 3–4)	3.30 ± 0.82 (Range: 2–4)
6. I thought there was too much inconsistency in this system	3.25 ± 0.50 (Range: 3–4)	3.50 ± 0.53 (Range: 3–4)
7. I would imagine that most people would learn to use this system very quickly	3.25 ± 0.50 (Range: 3–4)	3.10 ± 0.99 (Range: 1–4)
8. I found the system very cumbersome to use	3.25 ± 0.96 (Range: 2–4)	3.20 ± 1.23 (Range: 0–4)
9. I felt very confident using the system	3.75 ± 0.50 (Range: 3–4)	3.50 ± 0.71 (Range: 2–4)
10. I needed to learn a lot of things before I could get going with this system	2.75 ± 1.26 (Range: 1–4)	2.70 ± 1.57 (Range: 0–4)

The listed statistics are mean ± standard deviation (SD).

Note: The transformed score of each item is ranged from 0 to 4. For the odd- and even-numbered items, higher scores are indicative of higher and lower agreement with the statement of the item, respectively.

In addition, for occupational therapists, the mean score of the self-designed questionnaire was 41.00 (Table 3), which indicated good user experiences. Moreover, the items of the self-designed questionnaire with the highest average scores were “the concentration level of the participant while using this MSR system”, “the placement of the camera”, “the design of the working platform”, “the ease of use of the user manual”, and “willingness to use this MSR system in the future” (Table 5). However, the items of the self-designed questionnaire with the lowest average scores were “the resolution of the videos and images” and “the ease of operating the two adjustable arms”. Furthermore, the most common request from the therapists was that the resolution of the AO videos be improved.

**Table 5:** Mean ratings on each item of the self-designed questionnaire in occupational therapists and patients with stroke.

	Occupational therapists (*n* = 4)	Patients with stroke (*n* = 10)
1. The concentration level of participant while using this MSR system	4.50 ± 0.58 (Range: 4–5)	4.00 ± 0.82 (Range: 3–5)
2. The placement of the screen	4.00 ± 0.00 (Range: 4)	4.10 ± 0.99 (Range: 2–5)
3. The placement of the camera	4.50 ± 0.58 (Range: 4–5)	4.30 ± 0.82 (Range: 3–5)
4. The height of the seat	4.00 ± 1.41 (Range: 2–5)	4.60 ± 0.52 (Range: 4–5)
5. The design of the working platform	4.50 ± 0.58 (Range: 4–5)	4.10 ± 0.99 (Range: 2–5)
6. The resolution of videos and images	3.25 ± 1.26 (Range: 2–5)	4.20 ± 1.03 (Range: 2–5)
7. The ease of operating the two adjustable arms	3.25 ± 0.96 (Range: 2–4)	4.40 ± 0.97 (Range: 2–5)
8. The ease of use of the computer interface	4.00 ± 0.82 (Range: 3–5)	4.30 ± 0.67 (Range: 3–5)
9. The ease of use of the user manual	4.50 ± 0.58 (Range: 4–5)	4.40 ± 0.97 (Range: 2–5)
10. Willingness to use this MSR system in the future	4.50 ± 1.00 (Range: 3–5)	4.40 ± 0.84 (Range: 3–5)

The listed statistics are mean ± standard deviation (SD).

Abbreviations: MSR, multi-mode stroke rehabilitation.

Note: The score of each item is ranged from 1 to 5. Higher scores are indicative of more concentrated for item 1, more appropriate for items 2 to 5, better resolution for item 6, easier for items 7 to 9, and more willing for item 10.

#### Patient experiences and feedback

For the patients, the mean score of the SUS was 80.00 (Table 3), and the percentages of the SUS rating scores above 70 and 85 were 80% and 20%, respectively, which indicated good and excellent usability. Furthermore, for the stroke participants, the items of the SUS with the highest average scores were “I think that I would like to use this system frequently” and “I thought the system was easy to use”. The item of the SUS with the lowest average score was “I think that I would need the support of a technical person to be able to use this system” (Table 4).

In addition, for stroke participants, the mean score of the self-designed questionnaire was 42.80 (Table 3), which indicated good user experiences. The item of the self-designed questionnaire with the highest average score was “the height of the seat”, and the item with the lowest average score was “the concentration level of the participant while using this MSR system” (Table 5). Furthermore, the most 2 common requests from the patients were that a touch screen monitor be used and that the distance between the monitor and the user be increased during digital MT.

## Discussion

In this study, we successfully developed an innovative system (i.e., MSR) by combining two promising interventions, digital AOT and MT, for patients with stroke. In the first part of this study, we developed the hardware and software of the MSR system. In the second part of the study, the usability of the system was evaluated by occupational therapists and patients with stroke.

We integrated two promising rehabilitation therapies, digital AOT and MT, into the MSR system with multi-mode training (i.e., multi-functions) to achieve a more eclectic approach for stroke rehabilitation. Unlike the current computerized or digital MT devices^17,19,21–23^, which only provide MT as one type of treatment intervention, this MSR system, which additionally features digital AOT, extends the utility and variety for treating patients with stroke. Furthermore, the MSR system provides 2 training modes of digital AOT, one with pre-recorded and one with self-recorded videos. The former is the same as conventional AOT, but the latter is more flexible than conventional AOT, for it allows self-recording of the videos of functional tasks to meet the needs of individual patients with stroke.

Additionally, this MSR system also provides 2 training modes of digital MT, namely, unilateral and bilateral MT, which indicate the type of mirror visual feedback (MVF). Although the mechanism of MVF-induced effects has not been fully confirmed^31^, the common rationale for using MVF is that it increases the activity in the primary motor cortex of the stationary hand^31–33^, possibly via the incongruence of visual and somatosensory inputs, to action observation and the mirror neuron network^34,35^. A previous study indicated that MVF during MT may activate the contralateral sensorimotor cortex to make the brain more symmetrical and balanced in the motor recovery process of patients with stroke^36^. Conventional MT is capable of providing only unilateral MVF because the images are reflected in a plain mirror. However, the digital MT of our system has been designed to provide both unilateral and bilateral MVF, which can also be combined with either unimanual or bimanual upper-limb exercise, to increase the variety of MVF. However, further studies are needed to investigate the treatment efficacy of unilateral and bilateral MVF combined with unimanual and bimanual movements in the use of this new MSR system and examine the electrophysiological mechanisms underlying different MVF conditions, such as by using electroencephalography. These study findings are anticipated to provide the insights into the strengths and weakness of unilateral and bilateral MVF.

In the second part of this study, our findings showed that this MSR system can be applied safely and is usable by patients with stroke with minimal assistance. Although the percentages of occupational therapists (75%) and patients (80%) who rated the SUS above 70 were slightly different after they used the system, the usability evaluation demonstrated that over three quarters of the therapists and patients considered the MSR system to have good usability. Notably, the occupational therapists and patients expressed that they had high confidence while using this MSR system, they thought the system was easy to use, and they would like to use this system frequently. In addition, according to the results of the self-designed questionnaire, both the occupational therapists and the patients had good user experiences. The average rating scores on the individual items of the self-designed questionnaire given by the therapists and the patients were all above 4, except for the two items reported by the therapists, namely, the resolution of the videos and images and the ease of operating the two adjustable arms. With the advancement of technology, the resolution of the video clips and images can be increased, and the operation of the adjustable monitor arms can also be made easier in the near future.

The key contribution of this study is the development of the MSR system, which, to the best of our knowledge, is the first to integrate digital MT and AOT into one system for stroke rehabilitation intervention. The study results also demonstrate that this MSR system has good usability and provides good user experiences for both therapists and patients with stroke. Such a multi-mode MSR system might be further used to complement conventional rehabilitation for two reasons: It can provide more access to rehabilitation programs for patients with stroke, and it is easy to use for therapists and patients.

Regarding the study limitations, the captured movements of digital MT and recorded movements of digital AOT in this MSR system were still constrained to more distal movements of the upper limbs due to the placement of the webcam and screen. In the usability evaluation, the use of a convenience sample of patients with stroke may have partially limited the generalizability of our findings, especially for those with severe upper-limb motor impairments. Other possible confounders, such as familiarity with computers or technology and the demographic and clinical characteristics of the participants (e.g., education level, motor severity, etc.), also might have impacted the results of the usability evaluation and user experiences. Lastly, due to the small sample size of this study and lack of therapy effects provided by this system, further evidence is warranted.

We have capitalized on recent advances in video-based therapy and image technology to develop this new MSR system, which integrates digital MT and AOT in one system. Based on the study results, the MSR system seems usable to occupational therapists and patients with stroke. After making changes in response to the participants’ common feedback and requests, further work is suggested to include a wider range of stroke patients in a larger-scale feasibility study.

## Data Availability

The datasets generated during and/or analysed during the current study are not publicly available due to the confidentiality issue but are available from the corresponding author on reasonable request.

## References

[1] Meschia JF (2014). Guidelines for the primary prevention of stroke: a statement for healthcare professionals from the American Heart Association/American Stroke Association. Stroke.

[2] Katan M, Luft A (2018). Global Burden of Stroke. Semin. Neurol..

[3] Truelsen T (2006). Stroke incidence and prevalence in Europe: A review of available data. Eur. J. Neurol..

[4] Rajsic S (2019). Economic burden of stroke: A systematic review on post-stroke care. Eur. J. Health Econ..

[5] Hesse S (2011). Combined transcranial direct current stimulation and robot-assisted arm training in subacute stroke patients: An exploratory, randomized multicenter trial. Neurorehabil. Neural. Repair.

[6] Kwakkel G, Kollen BJ, van der Grond J, Prevo AJ (2003). Probability of regaining dexterity in the flaccid upper limb: Impact of severity of paresis and time since onset in acute stroke. Stroke.

[7] Stoykov, M. E. & Madhavan, S. Motor Priming in Neurorehabilitation. *J. Neurol. Phys. Ther.* **39** (2015). 10.1097/NPT.0000000000000065 PMC4270918 25415551

[8] Ramachandran VS, Rogers-Ramachandran D, Cobb S (1995). Touching the phantom limb. Nature.

[9] Thieme, H., Mehrholz, J., Pohl, M., Behrens, J. & Dohle, C. Mirror therapy for improving motor function after stroke. *Cochrane Database Syst. Rev.* **3** (2012). 10.1002/14651858.CD008449.pub2 PMC6464769 22419334

[10] Buccino G (2014). Action observation treatment: A novel tool in neurorehabilitation. Philos. Trans. R Soc. Lond. B Biol. Sci..

[11] Peng TH (2019). Action observation therapy for improving arm function, walking ability, and daily activity performance after stroke: A systematic review and meta-analysis. Clin Rehabil.

[12] Zhang, B. *et al.* The effects of action observation training on improving upper limb motor functions in people with stroke: A systematic review and meta-analysis. *PLoS One* **14**, e0221166 (2019). 10.1371/journal.pone.0221166 PMC6716645 31469840

[13] Zeng W, Guo Y, Wu G, Liu X, Fang Q (2018). Mirror therapy for motor function of the upper extremity in patients with stroke: a meta-analysis. J Rehabil Med.

[14] Thieme, H. *et al.* Mirror therapy for improving motor function after stroke. *Cochrane Database Syst Rev* **7**, CD008449 (2018). 10.1002/14651858.CD008449.pub3 PMC6513639 29993119

[15] Kim J, Yi J, Song CH (2017). Kinematic analysis of head, trunk, and pelvic motion during mirror therapy for stroke patients. J. Phys. Ther. Sci..

[16] Mehnert, J., Brunetti, M., Steinbrink, J., Niedeggen, M. & Dohle, C. Effect of a mirror-like illusion on activation in the precuneus assessed with functional near-infrared spectroscopy. *J Biomed Opt* **18**, 066001 (2013). 10.1117/1.JBO.18.6.066001 PMC4023640 23733017

[17] Ding L (2018). Camera-Based Mirror Visual Feedback: Potential to Improve Motor Preparation in Stroke Patients. IEEE Trans. Neural Syst. Rehabil. Eng..

[18] Giraux P, Sirigu A (2003). Illusory movements of the paralyzed limb restore motor cortex activity. Neuroimage.

[19] Hoermann S (2017). Computerised mirror therapy with Augmented Reflection Technology for early stroke rehabilitation: Clinical feasibility and integration as an adjunct therapy. Disabil. Rehabil..

[20] Laver KE (2018). Virtual reality for stroke rehabilitation. Stroke.

[21] Lee D, Lee M, Lee K, Song C (2014). Asymmetric training using virtual reality reflection equipment and the enhancement of upper limb function in stroke patients: A randomized controlled trial. J. Stroke Cerebrovasc..

[22] Lee H, Li P, Fan S (2015). Delayed mirror visual feedback presented using a novel mirror therapy system enhances cortical activation in healthy adults. J. NeuroEng. Rehabil..

[23] Weber LM, Nilsen DM, Gillen G, Yoon J, Stein J (2019). Immersive virtual reality mirror therapy for upper limb recovery after stroke: A pilot study. Am. J. Phys. Med. Rehabil0.

[24] Mekbib DB (2020). Proactive motor functional recovery following immersive virtual reality-based limb mirroring therapy in patients with subacute stroke. Neurotherapeutics.

[25] Chang CS (2019). Alternative motor task-based pattern training with a digital mirror therapy system enhances sensorimotor signal rhythms post-stroke. Front. Neurol..

[26] Dohle C, Kleiser R, Seitz RJ, Freund HJ (2004). Body scheme gates visual processing. J. Neurophysiol..

[27] Fugl-Meyer AR, Jaasko L, Leyman I, Olsson S, Steglind S (1975). The post-stroke hemiplegic patient: 1: A method for evaluation of physical performance. Scand. J. Rehabil. Med..

[28] Lum PS, Burgar CG, Shor PC, Majmundar M, Van der Loos M (2002). Robot-assisted movement training compared with conventional therapy techniques for the rehabilitation of upper-limb motor function after stroke. Arch. Phys. Med. Rehabil..

[29] Brooke, J. in *Usability evaluation in industry* (ed Jordan, P.W.) 189–194 (Taylor & Francis, 1996).

[30] Bangor, A., Kortum, P. T. & Miller, J. T. An empirical evaluation of the system usability scale. *Int J Hum Comput Interact* 574–594 (2008).

[31] Nojima I (2012). Human motor plasticity induced by mirror visual feedback. J. Neurosci..

[32] Garry MI, Loftus A, Summers JJ (2005). Mirror, mirror on the wall: viewing a mirror reflection of unilateral hand movements facilitates ipsilateral M1 excitability. Exp. Brain Res..

[33] Tominaga W (2009). A mirror reflection of a hand modulates stimulus-induced 20-Hz activity. Neuroimage.

[34] Zhang JJQ, Fong KNK, Welage N, Liu KPY (2018). The activation of the mirror neuron system during action observation and action execution with mirror visual feedback in stroke: a systematic review. Neural Plast.

[35] Saleh S, Yarossi M, Manuweera T, Adamovich S, Tunik E (2017). Network interactions underlying mirror feedback in stroke: A dynamic causal modeling study. Neuroimage Clin..

[36] Fong KNK, Ting KH, Chan CCH, Li LSW (2019). Mirror therapy with bilateral arm training for hemiplegic upper extremity motor functions in patients with chronic stroke. Hong Kong Med. J..

